# Determination of a “Specific Population Who Could Benefit From Rosuvastatin”: A Secondary Analysis of a Randomized Controlled Trial to Uncover the Novel Value of Rosuvastatin for the Precise Treatment of ARDS

**DOI:** 10.3389/fmed.2020.598621

**Published:** 2020-11-27

**Authors:** Shi Zhang, Zhonghua Lu, Zongsheng Wu, Jianfeng Xie, Yi Yang, Haibo Qiu

**Affiliations:** Jiangsu Provincial Key Laboratory of Critical Care Medicine, Department of Critical Care Medicine, School of Medicine, Nanjing Zhongda Hospital, Southeast University, Nanjing, China

**Keywords:** ARDS, Rosuvastatin, heterogeneity, machine learning, precise treatment

## Abstract

**Background:** The high heterogeneity of acute respiratory distress syndrome (ARDS) contributes to paradoxical conclusions from previous investigations of rosuvastatin for ARDS. Identification of the population (phenotype) that could benefit from rosuvastatin is a novel exploration for the precise treatment.

**Methods:** The patient population for this analysis consisted of unique patients with ARDS enrolled in the SAILS trial (rosuvastatin vs. placebo). Phenotypes were derived using consensus k-means clustering applied to routinely available clinical variables within 6 h of hospital presentation before the patients received placebo or rosuvastatin. The Kaplan–Meier statistic was used to estimate the 90-day cumulative mortality to screen for a specific population that could benefit from rosuvastatin, with a cutoff *P* < 0.05.

**Results:** The derivation cohort included 585 patients with ARDS. Of the patients with the four derived phenotypes, those with phenotype 3 were classified as the “specific population who could benefit from rosuvastatin” as rosuvastatin resulted in a significant reduction in 90-day cumulative mortality from ARDS [hazard ratio (HR), 0.29; 95% confidence interval (CI), 0.09–0.93; *P* = 0.027]. Additionally, rosuvastatin markedly improved the days free of cardiovascular failure (10.08 ± 3.79 in the rosuvastatin group vs. 7.31 ± 4.94 in the placebo group, *P* = 0.01) and coagulation abnormalities (13.65 ± 1.33 vs. 12.15 ± 3.77, *P* = 0.02) up to day 14 in the phenotype 3 cohort. Phenotype 3 was summarized as Platelet^high^ & Creat^low^ phenotype because these patients have a relatively higher platelet count (390.05 ± 79.43 × 10^9^/L) and lower creatinine (1.42 ± 1.08 mg/dL) than do patients classified as other phenotypes. In addition, rosuvastatin seemed to increase 90-day mortality for patients classified as phenotype 4 (HR, 2.76; 95% CI, 0.09–9.93; *P* = 0.076), with an adverse effect on reducing the days free of renal failure up to day 14 (4.70 ± 4.99 vs. 10.17 ± 4.69, *P* = 0.01). Patients in phenotype 4 showed relatively severe illness in terms of baseline features, particularly renal failure, with high serum glucose. Therefore, phenotype 4 was defined as APACHE^high^ & Serum glucose^high^ phenotype.

**Conclusions:** This secondary analysis of the SAILS trial identified that rosuvastatin seems to be harmful for patients classified as APACHE^high^ & Serum glucose^high^ phenotype, but benefit patients in Platelet^high^ & Creat^low^ phenotype, thus uncovering the novel value of rosuvastatin for the precise treatment of ARDS.

## Background

Acute respiratory distress syndrome (ARDS) is a highly heterogeneous and complicated critical illness. Despite advances in clinical management, the mortality rate of severe ARDS remains as high as 40–46% because of the lack of targeted therapeutic protocols for distinct patients. Categorizing ARDS for further appropriate therapy is a critical unmet need for precise treatment and improvement of the salvage rate of ARDS (1, 2).

In consideration of rosuvastatin's anti-inflammatory effects and pathogenesis of ARDS (inadequate control of inflammatory responses in the lung), rosuvastatin has been utilized in the treatment of ARDS in the last decade (3–7). Previous studies demonstrated that rosuvastatin could improve the outcomes of ARDS in animal models (8–10). Unfortunately, a large multicenter randomized controlled trial conducted in 2014 by Truwit et al. (named the SAILS trial) suggested that rosuvastatin therapy did not improve the clinical outcomes of patients with ARDS (11).

A possible reason for these paradoxical conclusions is the heterogeneity of ARDS. ARDS, as an overly broad definition of a syndrome, encompasses a vast, multidimensional array of clinical and biological features. Markedly different from experimental animals, patients with ARDS actually comprise diverse phenotypes, which appear to have different clinical characteristics, immune statuses, biological processes, and severities. Several investigations successfully classified ARDS into distinct subgroups via biomarkers or clinical features (12, 13) and indicated that appropriate therapies for distinct patients may be a promising strategy for precise treatment in ARDS. Rosuvastatin, as an immunomodulatory intervention to attenuate inflammation, may benefit only some specific populations. Although Sinha et al. (14) conducted a latent class analysis of ARDS subphenotypes in the SAILS trial, the subphenotype that can benefit from rosuvastatin was not identified in their analysis. The reason for this may be that Sinha et al. did not utilize a matched algorithm and appropriate data processing for their data. Obviously, there is a robust need to identify the treatable ARDS phenotype (patients who could benefit from rosuvastatin) through a large number of various algorithms and data analyses.

Fortunately, Truwit et al. (11) uploaded the original data of the SAILS trial to the ARDS-Net database, making it possible for us to perform a secondary analysis to find the specific population that could benefit from rosuvastatin. Thus, we aimed to derive this specific ARDS phenotype by using an unsupervised clustering algorithm to uncover the novel value of rosuvastatin for the precise treatment of ARDS.

## Methods

This study was reviewed and approved by the Institutional Ethics Committee of Zhongda Hospital. The Institutional Ethics Committee of Zhongda Hospital approved this study, which was conducted under several data use agreements. The data for the ARDSnet project were obtained under a waiver of informed consent and with authorization under the Health Insurance Portability and Accountability Act.

### Patient Population

The patient population for this analysis consisted of unique patients with ARDS enrolled in the SAILS trial (rosuvastatin vs. placebo), which was published in 2014. The diagnostic criterion of ARDS in the SAILS trial referenced the 2012 Berlin definition of ARDS (1, 2). To eliminate the influence of immunosuppression on the evaluation of rosuvastatin for ARDS, the patients were divided into 160 definitely immunosuppressed patients and 585 other patients for the respective analysis. The definitely immunosuppressed patients included ARDS patients with comorbidities such as acquired immune deficiency syndrome, leukemia, and non-Hodgkin lymphoma; patients with cancer receiving chemotherapy; and patients who received immunosuppression therapy in the past 6 months. After excluding the 160 definitely immunosuppressed patients, 585 other patients were enrolled in the derivation cohort for further unsupervised clustering analysis.

### Screening Clinical Features for Phenotyping

Based on the SAILS trial database, we first extracted the available variables within the first 6 h of hospital presentation before the patients received placebo or rosuvastatin and excluded variables with missing rates > 10%. These clinically available characteristics included age, alanine aminotransferase, APACHE III score, aspartate aminotransferase, blood urea nitrogen, C-reactive protein, creatine kinase, creatinine, diastolic blood pressure (BP), Glasgow Coma Scale score, height, heart rate, male sex, Paco_2_, Pao_2_:Fio_2_, Pao_2_, platelet count, predicted body weight, respiration rate, serum albumin highest, serum albumin lowest, serum glucose lowest, shock at baseline, systolic BP, temperature, urine output, and weight.

Furthermore, to screen the candidate variables that could identify a “specific population who can benefit from rosuvastatin,” we conducted differential analyses by using *t*-tests to compare clinically available variables between the rosuvastatin group and placebo group among surviving patients, and *P* < 0.3 was the threshold value.

### Statistical Methods

To derive the phenotypes, we first assessed the candidate variable distributions, missingness, and correlation. Multiple imputations with chained equations were used to account for missing data (15).

To identify different phenotypes of ARDS, consensus k-means clustering through candidate variables was utilized to perform consistent clustering on 585 patients in the derivation cohort (16). Clustering was performed using 100 iterations, with each iteration containing 80% of the samples. The optimal clustering strategy was determined by cumulative distribution function curves of the consensus score, clear separation of the consensus matrix heatmaps, characteristics of the consensus cumulative distribution function plots, and adequate pairwise-consensus values between cluster members.

To evaluate the effect of rosuvastatin on the outcomes of ARDS in different subgroups, Kaplan–Meier statistics were used to estimate 90-day mortality. Organ failure–free days up to day 14 (day), days free of cardiovascular failure up to day 14 (day), days free of coagulation abnormality to up day 14 (day), days free of hepatic failure up to day 14 (day), days free of renal failure up to day 14 (day), intensive care unit–free days to up day 28 (day), and ventilator-free days to up day 28 were analyzed by means of analysis of variance. Twenty-eight-day mortality, 60-day mortality, and 90-day mortality were analyzed by the χ^2^ test. *P* < 0.05 was set as the threshold value to screen for significant results.

To observe the clinical feature variations among different phenotypes, the means of analysis of variance and χ^2^ tests were utilized to assess continuous variables and dichotomous variables, respectively, with a cutoff value of *P* < 0.05.

Brief flow plots of these analyses are shown in Supplementary Figure 1.

### Software and Versions

R × 64 3.6.1 was applied to process the data, analyze the data, and plot diagrams.

## Results

### Patients

A total of 745 patients who met the ARDS criteria were enrolled in the final analysis, with 379 patients in the rosuvastatin group and 366 patients in the placebo group. The age of the investigated patients ranged from 18 to 89 (median, 54), and 51% were male. The mean Pao_2_:Fio_2_ level was 143.48 mmHg (standard deviation [SD], 63.57 mmHg), and the mean APACHE III score was 93.42 (SD, 20.15 mmHg). The detailed baseline demographic and clinical characteristics are shown in Supplementary Tables 1, 2.

### Derivation of ARDS Phenotypes

After a differential analysis of the clinically available variables, we finally found that the highest serum glucose, C-reactive protein, and platelet count were candidate variables for further unsupervised clustering analysis, as shown in Supplementary Table 3.

**Figure 1:**
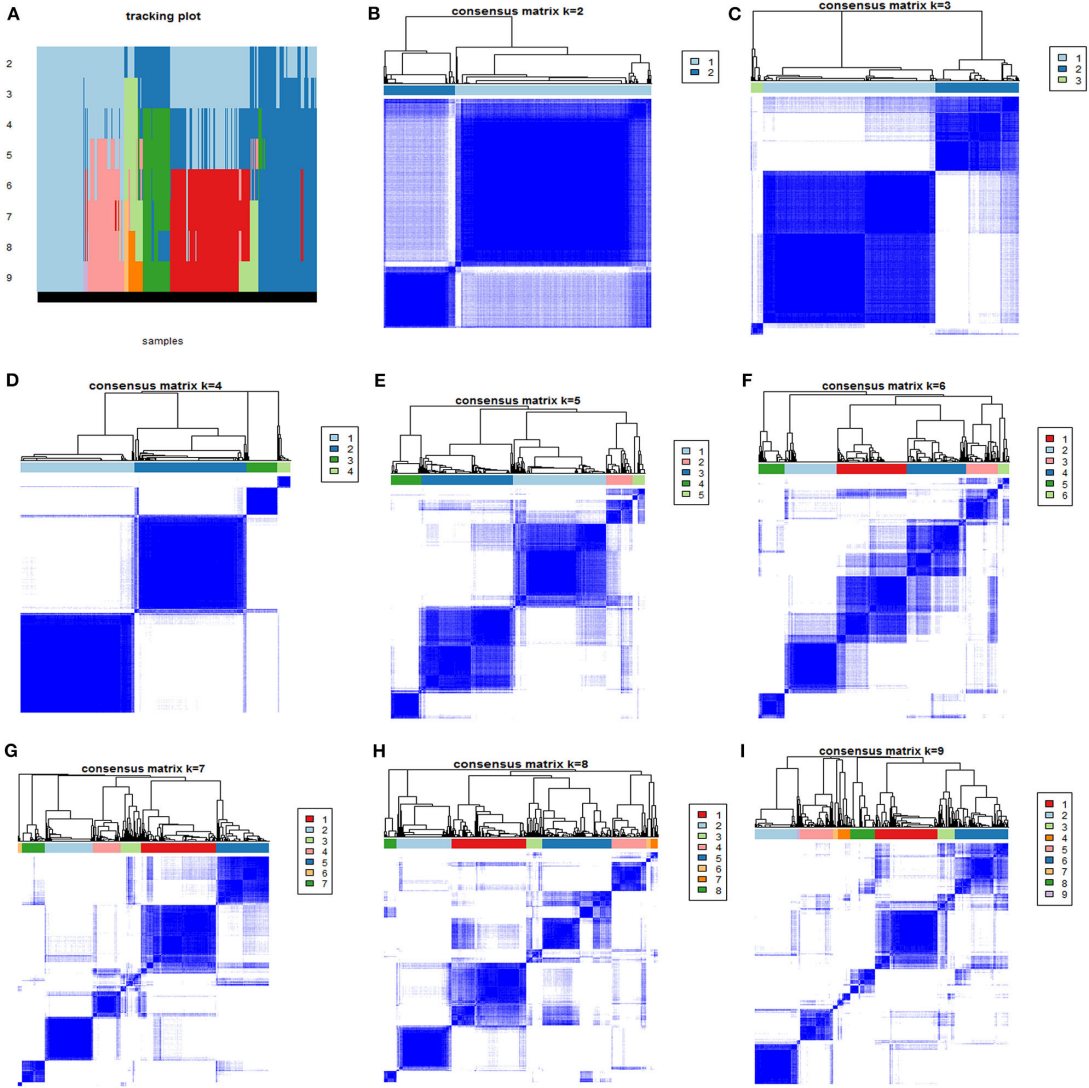
The consensus matrix heatmaps of consensus k-means clustering. **(A)** Shows the sample distribution of 4 phenotypes after consensus k-means clustering. **(B–I)** Shows consensus matrix heatmaps of different subgroup numbers (*k* = 2, 3, 4, 5, 6, 7, 8, and 9). When *k* = 4, the model exhibited the clearest separation of the consensus matrix heatmap.

After excluding the 160 definitely immunosuppressed patients, 585 patients were enrolled in the derivation cohort. The consensus k-means clustering models suggested that a four-class model was the optimal fit for the four phenotypes, as the clearest separation of the consensus matrix heatmap could be found in the four-class model, as shown in Figure 1.

### Patients Classified as Platelet^high^ & Creat^low^ Phenotype Could Benefit From Rosuvastatin

**Figure 2:**
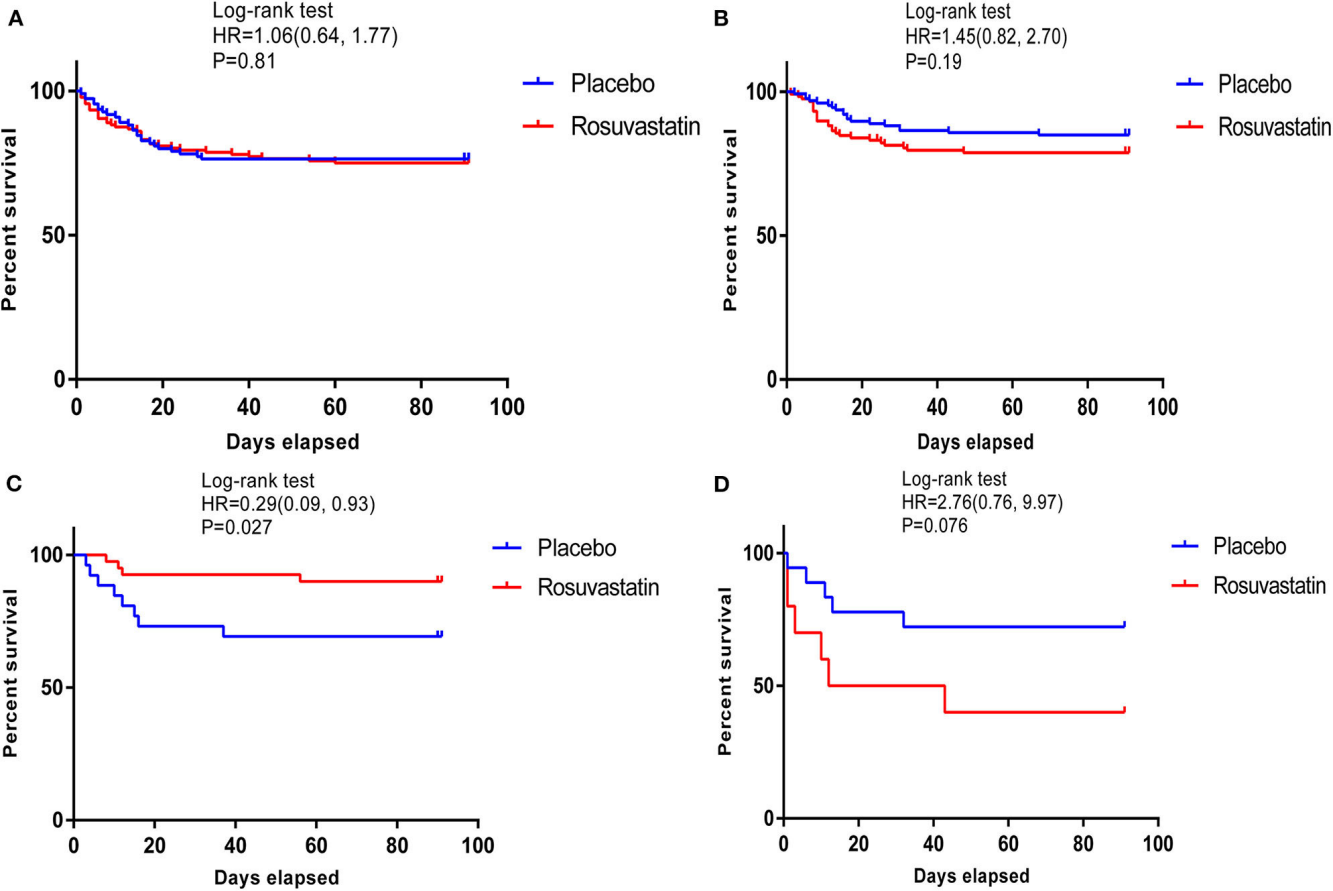
Kaplan–Meier survival curves of 90-day cumulative mortality for patients receiving rosuvastatin and patients receiving placebo among those with the 4 phenotypes. **(A–D)** shows survival curves of the patients with phenotypes 1–4. In the phenotype 3 cohort, rosuvastatin resulted in a significant reduction in 90-day cumulative mortality [hazard ratio [HR] 0.29 [95% CI 0.09, 0.93]; *P* = 0.027].

According to Kaplan–Meier statistical analysis, the phenotype 3 cohort was identified as the “specific population who can benefit from rosuvastatin,” as shown in Figure 2. In the phenotype 3 cohort, rosuvastatin resulted in a significant reduction in cumulative 90-day mortality from ARDS [hazard ratio (HR), 0.29; 95% confidence interval (CI), 0.09–0.93; *P* = 0.027]. Moreover, there were no significant differences in the baseline characteristics between those assigned to rosuvastatin and those assigned to placebo in the phenotype 3 cohort. The baseline characteristics of the patients with the four derived phenotypes are shown in Supplementary Tables 4–7.

In the phenotype 3 cohort, the days free of cardiovascular failure and coagulation abnormalities up to day 14 differed significantly between the patients who received rosuvastatin and those who received placebo. Additionally, rosuvastatin resulted in a slight increase in ventilator-free days up to day 28 for patients with ARDS. There were no significant between-group differences in any of the other outcomes. The above results are presented in Table 1.

**Table 1:** Outcomes in different phenotypes.

**Outcomes**	**Placebo**	**Rosuvastatin**	***P***
**28 day mortality (%)**			
Phenotype 1	23%	21%	0.70
Phenotype 2	12%	17%	0.20
Phenotype 3	27%	14%	0.07
Phenotype 4	22%	50%	0.28
**60 day mortality (%)**			
Phenotype 1	24%	25%	1
Phenotype 2	14%	21%	0.21
Phenotype 3	31%	10%	0.07
Phenotype 4	28%	32%	0.20
**90 day mortality (%)**			
Phenotype 1	24%	25%	1
Phenotype 2	15%	21%	0.28
Phenotype 3	31%	10%	0.07
Phenotype 4	28%	32%	0.20
**Organ failure free days to day 14(day)**			
Phenotype 1	6.16 ± 5.14	6.31 ± 5.32	0.83
Phenotype 2	8.39 ± 5.04	8.21 ± 5.16	0.79
Phenotype 3	7 ± 5.23	8.83 ± 4.62	0.14
Phenotype 4	6.72 ± 5.13	3 ± 4.62	0.07
**Free of cardiovascular failure to day14 (day)**			
Phenotype 1	10.37 ± 4.80	10.72 ± 4.93	0.57
Phenotype 2	9.81 ± 4.44	9.19 ± 4.67	0.29
Phenotype 3	7.31 ± 4.94	10.08 ± 3.79	0.01
Phenotype 4	7.94 ± 4.99	6.20 ± 5.51	0.40
**Free of coagulation abnormality to day14 (day)**			
Phenotype 1	10.83 ± 5.12	14.93 ± 9.80	0.38
Phenotype 2	13.30 ± 2.22	12.90 ± 2.81	0.21
Phenotype 3	12.15 ± 3.77	13.65 ± 1.33	0.02
Phenotype 4	10.67 ± 5.10	8.10 ± 6.10	0.24
**Free of hepatic failure to day 14 (day)**			
Phenotype 1	11.06 ± 4.65	9.89 ± 5.36	0.07
Phenotype 2	13.29 ± 2.46	12.51 ± 3.38	0.04
Phenotype 3	11.81 ± 4.17	12.83 ± 3.01	0.25
Phenotype 4	11.50 ± 4.85	7.70 ± 6.41	0.09
**Free of renal failure to day 14 (day)**			
Phenotype 1	10.50 ± 4.88	11.45 ± 4.25	0.41
Phenotype 2	11.74 ± 4.22	11.44 ± 4.64	0.60
Phenotype 3	10.50 ± 4.88	11.45 ± 4.25	0.41
Phenotype 4	10.17 ± 4.69	4.70 ± 4.99	0.01
**ICU free days to day 28 (day)**			
Phenotype 1	13.82 ± 9.83	14.93 ± 9.80	0.38
Phenotype 2	17.05 ± 9.07	15.74 ± 9.72	0.27
Phenotype 3	12.96 ± 11.38	17.35 ± 8.59	0.08
Phenotype 4	13 ± 10.45	9 ± 10.50	0.34
**Ventilator free days to day 28 (day)**			
Phenotype 1	14.17 ± 10.90	15.43 ± 10.62	0.36
Phenotype 2	18.07 ± 9.68	17.02 ± 10.12	0.41
Phenotype 3	13.27 ± 11.90	18.75 ± 8.93	0.04
Phenotype 4	13.67 ± 11.58	10.1 ± 11.47	0.44

*Organ failure free days to day 14: No. of days without failure of circulatory, coagulation, hepatic, or renal organs from Day 1 to 14*.

For better insight into the patients who could benefit from rosuvastatin, we compared the clinical characteristics among different phenotypes. Phenotype 3 was summarized as Platelet^high^ & Creat^low^ phenotype because patients in this phenotype have a relatively higher platelet count (390.05 ± 79.43 × 10^9^/L) and lower creatinine (1.42 ± 1.08 mg/dL) than patients classified as other phenotypes. Additionally, the other distinct clinical characteristics of the patients with different phenotypes are described in Table 2. Indeed, phenotype 3 could be identified through our four-class model.

**Table 2:** Clinical characteristics variations in different phenotypes.

**Characteristics**	**Phenotype 1**	**Phenotype 2**	**Phenotype 3**	**Phenotype 4**	***P***
	**(*n =* 247)**	**(*n =* 244)**	**(*n =* 66)**	**(*n =* 66)**	
Age (year)	54.07 ± 16.72	53.83 ± 16.96	54.52 ± 16.64	56.79 ± 13.94	0.84
Male, No. %	48%	51%	55%	50%	0.78
Weight (kg)	85.94 ± 28.25	92.23 ± 34.78	92.85 ± 30.63	87.36 ± 28.59	0.12
Height (kg)	168.83 ± 10.17	168.89 ± 11.41	169.46 ± 10.68	170.39 ± 13.26	0.88
Predicted Body Weight (kg)	62.74 ± 10.87	62.61 ± 11.97	63.03 ± 11.18	64.11 ± 13.81	0.93
APACHE III	95.47 ± 28.60	83.61 ± 24.97	87.69 ± 27.28	110.18 ± 24.35	<0.01
Temperature (°C)	37.31 ± 0.97	37.45 ± 0.98	37.52 ± 0.95	37.82 ± 0.86	0.04
Shock, No. %	63%	47%	55%	68%	<0.01
Respiratory rate	25.10 ± 7.20	25.52 ± 7.11	24.95 ± 6.03	24.64 ± 5.69	0.84
Pao_2_ (mmHg)	91.21 ± 33.70	90.17 ± 31.78	95.06 ± 43.23	113.64 ± 46.30	<0.01
Paco_2_ (mmHg)	38.24 ± 9.45	41.67 ± 9.99	41.29 ± 9.20	35.71 ± 9.03	<0.01
Pao_2_:Fio_2_ (mmHg)	139.78 ± 61.86	148.64 ± 62.31	139.27 ± 65.47	128.61 ± 76.91	0.24
Heart rate (beats/min)	96.25 ± 19.57	95.17 ± 19.05	94.97 ± 18.84	102 ± 21.06	0.34
Systolic BP (mmHg)	109.77 ± 18.98	114.72 ± 18.49	115.02 ± 19.37	108.11 ± 19.13	0.01
Diastolic BP (mmHg)	60.63 ± 11.76	61.37 ± 13.93	60.89 ± 14.22	55.35 ± 10.30	0.14
Glasgow Coma Scale	7.60 ± 3.24	8.24 ± 3.51	7.92 ± 3.56	6.46 ± 3.33	0.03
Alanine aminotransferase (U/liter)	49.32 ± 8.91	47.85 ± 8.83	49.03 ± 10.19	46.86 ± 10.59	0.23
Aspartate aminotransferase (U/liter)	41.72 ± 4.32	42.10 ± 5.20	41.29 ± 5.69	40.89 ± 5.95	0.45
Urine output within 24 h of hospital presentation	1,457 ± 1,211	1,740 ± 1,253	1,830 ± 1,380	1,456 ± 1,106	0.03
Blood urea nitrogen (mmol/L)	27.40 ± 19.63	24.63 ± 17.68	24.29 ± 18.11	38.04 ± 28.59	<0.01
Creatine kinase (U/liter)	244.63 ± 51.68	241.45 ± 53.39	233.29 ± 55.59	226.5 ± 56.79	0.20
Creat (mg/dl)	1.65 ± 1.28	1.47 ± 1.10	1.42 ± 1.08	2.25 ± 1.32	<0.01
Serum Glucose Highest (mg/dL)	148.36 ± 50.32	152.57 ± 49.78	157.21 ± 47.07	484.35 ± 154.83	<0.01
Serum Glucose Lowest (mg/dL)	114.44 ± 39.74	125.02 ± 40.96	125.56 ± 39.65	186.11 ± 116.62	<0.01
Serum Albumin Highest (g/dL)	2.24 ± 0.74	2.43 ± 0.63	2.20 ± 0.73	2.46 ± 0.88	<0.01
Serum Albumin Lowest (g/dL)	2.18 ± 0.69	2.36 ± 0.61	2.11 ± 0.70	2.36 ± 0.78	<0.01
Platelet count (10^9^/L)	103.79 ± 39.97	222.22 ± 41.66	390.05 ± 79.43	176.68 ± 94.30	<0.01
CRP (μg/L)	26.04 ± 34.69	28.69 ± 27.96	20.23 ± 11.99	26.25 ± 13.92	0.22

### Rosuvastatin Seems to Be Harmful for Patients Classified as APACHE^high^ & Serum Glucose^high^ Phenotype

The survival curves of phenotype 4 illuminated a trend that rosuvastatin resulted in a reduction in the 90-day survival rate of ARDS, despite the less rigorous confidence interval (HR, 2.76; 95% CI, 0.09–9.93; *P* = 0.076). Patients in phenotype 4 showed the early renal failure, with the highest APACHE III score (110.18 ± 24.35), blood urea nitrogen (38.04 ± 28.59 mmol/L), creatinine (2.25 ± 1.32 mg/dL), serum glucose (484.35 ± 154.83 mg/dL), and morbidity of shock at baseline (68%) and the lowest Pao_2_:Fio_2_ (128.61 ± 76.91 mmHg) and Glasgow Coma Scale score (6.46 ± 3.33). Therefore, phenotype 4 was summarized as APACHE^high^ & Serum glucose^high^ phenotype.

### Characteristics and Outcomes of Patients With Other Phenotypes

Kaplan–Meier survival analysis indicated that rosuvastatin had no effect on ARDS in the cohorts with the other phenotypes. In the phenotype 2 cohort, rosuvastatin appeared to slightly reduce the days free of hepatic failure up to day 14. In addition, rosuvastatin led to a moderate reduction in the days free of renal failure up to day 14 in the phenotype 4 cohort. More details of the characteristics and outcomes of the patients with other phenotypes are described in Tables 1, 2.

The survival curves of the patients with the four phenotypes are shown in Supplementary Figure 2, and the survival curves of definitely immunosuppressed patients are shown in Supplementary Figure 3.

## Discussion

In this secondary analysis of the SAILS trial, four phenotypes of ARDS were derived through routinely available clinical variables at the time of hospital presentation. These phenotypes were multidimensional, and the patients were heterogeneous in their demographics, clinical characteristics, several laboratory abnormalities, and effects of rosuvastatin therapy; these phenotypes differed from traditional patient classifications such as those based on direct or indirect lung injury, patterns of organ dysfunction, or severity of ARDS. In the phenotype 3 cohort, rosuvastatin exhibited benefits for patients with ARDS compared with placebo. This conclusion highlights the importance of characterizing the heterogeneity of ARDS and early goal-directed therapy.

To the best of our knowledge, the current study is the first to identify a specific population that can benefit from rosuvastatin, which could improve the therapeutic strategies for ARDS and reduce mortality. Furthermore, validation clinical trials are warranted to further assess these factors. These patients exhibited relatively higher platelet counts (390.05 ± 79.43 × 10^9^/L) and lower creatinine (1.42 ± 1.08 mg/dL) levels than other patients with ARDS, thus summarized as Platelet^high^ & Creat^low^ phenotype. These patients probably suffered from a relatively slight infection and might benefit from rosuvastatin because its anti-inflammatory effect could rapidly restore cardiovascular function. Indeed, the current study indicated that rosuvastatin resulted in an obvious improvement in days free of cardiovascular failure up to day 14 (7.31 ± 4.94 in placebo vs. 10.08 ± 3.79 in rosuvastatin, *P* = 0.01). Phenotype 3 could be rapidly identified through our machine learning–constructed four-class model. This model could be utilized to identify specific populations who can benefit from rosuvastatin at the time of patient presentation to the emergency department and thus could be useful with regard to early treatment and enrollment in clinical trials. Only routinely available data were used in the clustering models, and the phenotypes were derived from a large observational cohort to ensure generalizability.

Rosuvastatin may improve inflammatory responses, possibly via modulation of a platelet-dependent mechanism, which might be a potential treatment pathogenesis of rosuvastatin for this novel phenotype for ARDS. It is well-known that platelets play an important role in neutrophil-mediated lung injury (17, 18). The present study indicated that patients classified as phenotype 3 exhibited relatively high platelet counts. Additionally, in these patients, rosuvastatin significantly improved the coagulation abnormalities of ARDS compared with placebo. Therefore, we hypothesized that platelets might be involved in the pharmacological mechanism of rosuvastatin in specific patients with ARDS, and validation experiments are warranted to assess these related mechanisms.

Rosuvastatin might be harmful for patients with definite immunosuppression. Rosuvastatin was previously utilized in patients with ARDS mainly because of rosuvastatin's anti-inflammatory effects. However, infection is the main risk factor for ARDS, and it has been verified that patients with immunosuppression had worse outcomes as their weak immune systems could barely eliminate the pathogens (19, 20). Therefore, the immunosuppressive effect of rosuvastatin could not benefit such patients. This study similarly exhibited a trend that patients with definite immunosuppression probably had a worse outcome when receiving rosuvastatin, as shown in Figure 1A.

Rosuvastatin seems to be harmful for patients classified as phenotype 4. The survival curves of phenotype 4 illuminated a trend that rosuvastatin resulted in a reduction in the 90-day survival rate of ARDS, despite the less rigorous confidence interval (HR, 2.76; 95% CI, 0.09–9.93; *P* = 0.076). Furthermore, the current analysis on days free of renal failure up to day 14 suggested that rosuvastatin might aggravate renal damage (10.17 ± 4.69 in the placebo group vs. 4.70 ± 4.99 in the rosuvastatin group, *P* = 0.01). Patients with phenotype 4 showed the highest APACHE III score (110.18 ± 24.35), blood urea nitrogen (38.04 ± 28.59 mmol/L), creatinine (2.25 ± 1.32 mg/dL), serum glucose (484.35 ± 154.83 mg/dL), and morbidity of shock at baseline (68%) and the lowest Pao_2_:Fio_2_ (128.61 ± 76.91 mmHg) and Glasgow Coma Scale score (6.46 ± 3.33), as well as other clinical variables. In brief, patients with phenotype 4 showed relatively severe illness according to their baseline features, particularly renal failure, with high serum glucose. Therefore, phenotype 4 was defined as APACHE^high^ & Serum glucose^high^ phenotype.

There are several limitations to the present study. Indeed, the current analysis on treatment × phenotype interactions is largely limited by sample size. Therefore, these novel proof-of-concept ARDS phenotypes should be incorporated prospectively in future study designs that subsequently validate the effect of rosuvastatin on ARDS (21). In addition, for the limitation of clinical correlation analysis, further basic experiments should be conducted to sequentially research the elaborate mechanisms of rosuvastatin for ARDS indicated by our analyses.

## Conclusion

This secondary analysis of the SAILS trial identified rosuvastatin seems to be harmful for patients classified as APACHE^high^ & Serum glucose^high^ phenotype, but benefit patients with Platelet^high^ & Creat^low^ phenotype, thus uncovering the novel value of rosuvastatin for the precise treatment of ARDS.

## Data Availability Statement

Publicly available datasets were analyzed in this study. This data can be found at: http://www.ardsnet.org/.

## Ethics Statement

This study was reviewed and approved by Institutional Ethics Committee of Zhongda Hospital. Institutional Ethics Committee of Zhongda Hospital and conducted under several data use agreements.

## Author Contributions

SZ and HQ had full access to all of the data in the study and take responsibility for their integrity and the accuracy of the data analysis. SZ and ZL performed the data process, statistical analysis, and preparation of the article for publication. All authors participated in writing the article and preparing the figures.

## Conflict of Interest

The authors declare that the research was conducted in the absence of any commercial or financial relationships that could be construed as a potential conflict of interest.
