# Supplementary material for: Determination of a “Specific Population Who Could Benefit From Rosuvastatin”: A Secondary Analysis of a Randomized Controlled Trial to Uncover the Novel Value of Rosuvastatin for the Precise Treatment of ARDS
Source: Front Med (Lausanne). 2020 Nov 27;7:598621. doi: 10.3389/fmed.2020.598621 (PMC7737567; doi:10.3389/fmed.2020.598621)
Supplement: Supplementary file 1 [file Data_Sheet_1.DOC]

Catalogue of Supplemental Material

| Title | Content | Page*e* |
| --- | --- | --- |
| e-Table 1 | Baseline Characteristics of definitely immunosuppressed patients | 2 |
| e-Table 2 | Baseline Characteristics of other patients | 3 |
| e-Table 3 | Screen Features for further unsupervised clustering analysis | 4 |
| e-Table 4 | Baseline Characteristics of patients in phenotype1 | 5 |
| e-Table 5 | Baseline Characteristics of patients in phenotype2 | 6 |
| e-Table 6 | Baseline Characteristics of patients in phenotype3 | 7 |
| e-Table 7 | Baseline Characteristics of patients in phenotype4 | 8 |
| e-Figure 1 | The analysis flow plots of this study | 10 |
| e-Figure 2 | The survival curves of 4 phenotypes | 11 |
| e-Figure 3 | The survival curves of definitely immunosuppressed patients and other patients | 12 |

e-Table 1. Baseline Characteristics of definitely immunosuppressed patients

| Characteristics | Placebo(n=85) | Rosuvastatin(n=75) | P |
| --- | --- | --- | --- |
| Age — yr | 54.35±16.39 | 53.84±17.03 | 0.81 |
| Male sex — no. /total no. | 57% | 53% | 0.81 |
| White race — no./total no. | 79% | 88% | 0.36 |
| Primary cause of lung injury  — no./total no. | 80% | 87% | 0.36 |
| Weight — kg | 76.35±21.80 | 85.77±25.97 | 0.01 |
| APACHE III score | 103.46±26.34 | 104.44±28.95 | 0.83 |
| Shock at baseline —no./total no. | 51% | 52% | 0.98 |
| Systolic BP (mmHg) | 111.05±17.21 | 114.25±19.91 | 0.28 |
| Diastolic BP (mmHg) | 58.78±11.41 | 62.60±14.12 | 0.06 |
| Tidal volume — ml/kg of predicted body weight | 6.82±0.15 | 6.44±0.11 | 0.05 |
| Minute ventilation — liters/min | 10.53±3.24 | 10.79±3.08 | 0.61 |
| Inspiratory plateau pressure  — cm of water | 14.75±5.12 | 15.64±4.61 | 0.25 |
| PEEP — cm of water | 9.29±3.62 | 9.60±3.41 | 0.58 |
| PaCO2 — mm Hg | 40.60±11.19 | 41.81±12.98 | 0.53 |
| PaO2:FiO2 — mm Hg | 157.48±75.41 | 132.33±47.50 | 0.01 |
| Creatinine — mg/dl | 1.55±1.-4 | 1.63±10.6 | 0.66 |
| Creatine kinase — U/liter | 86.76±117.46 | 112.52±193.04 | 0.30 |
| Alanine aminotransferase  — U/liter | 28.84±21.90 | 43.30±59.18 | 0.04 |
| Aspartate aminotransferase  — U/liter | 40.65±34.86 | 68.96±3.20 | 0.06 |

e-Table 2. Baseline Characteristics of other patients

| Characteristics | Placebo(n=281) | Rosuvastatin(n=304) | P |
| --- | --- | --- | --- |
| Age — yr | 53.79±15.77 | 54.48±17.47 | 0.62 |
| Male sex — no. /total no. | 49% | 51% | 0.65 |
| White race — no./total no. | 87% | 85% | 0.84 |
| Primary cause of lung injury  — no./total no. | 77% | 80% | 0.32 |
| Weight — kg | 89.84±32.31 | 89.08±30.75 | 0.77 |
| APACHE III score | 92.13±27.82 | 88.85±27.47 | 0.16 |
| Shock at baseline —no./total no. | 56% | 55% | 0.88 |
| Systolic BP (mmHg) | 113.17±19.17 | 111.58±18.76 | 0.31 |
| Diastolic BP (mmHg) | 61.32±13.57 | 60.15±12.36 | 0.27 |
| Tidal volume — ml/kg of predicted body weight | 7.40±13.39 | 6.62±1.18 | 0.31 |
| Minute ventilation — liters/min | 10.98±3.10 | 10.78±3.02 | 0.42 |
| Inspiratory plateau pressure  — cm of water | 15.03±4.53 | 14.88±4.64 | 0.68 |
| PEEP — cm of water | 9.19±3.96 | 9.14±3.67 | 0.88 |
| PaCO2 — mm Hg | 41.77±14.63 | 40.85±11.71 | 0.39 |
| PaO2:FiO2 — mm Hg | 142.58±64.12 | 143.15±62.64 | 0.91 |
| Creatinine — mg/dl | 1.56±1.09 | 1.59±1.28 | 0.76 |
| Creatine kinase — U/liter | 252.79±440.01 | 251.65±451.55 | 0.98 |
| Alanine aminotransferase  — U/liter | 37.77±36.67 | 35.27±28.41 | 0.36 |
| Aspartate aminotransferase  — U/liter | 52.46±46.29 | 50.30±36.42 | 0.53 |

e-Table 3. Screen Features for further unsupervised clustering analysis

| Features | Placebo(n=270) | Treat(n=271) | P |
| --- | --- | --- | --- |
| Serum Glucose Highest (mg/dL) | 172 ± 5.67 | 152.1 ± 3.47 | <0.01 |
| C-reactive protein (μg/L) | 24.12 ± 1.10 | 27.22 ± 2.10 | 0.19 |
| Platelet (10^9/L) | 185.7 ± 6.44 | 195.5 ± 6.64 | 0.29 |
| APACHE III score | 89.6 ± 1.64 | 87.31 ± 1.62 | 0.32 |
| PaO2:FiO2 mmHg | 149.6 ± 4.2 | 144.1 ± 3.71 | 0.33 |
| Blood urea nitrogen (mmol/L) | 26.18 ± 1.15 | 24.61 ± 1.12 | 0.33 |
| Weight (kg) | 86.29 ± 1.76 | 88.64 ± 1.77 | 0.35 |
| Glasgow Coma Scale | 7.97 ± 0.21 | 8.21 ± 0.21 | 0.41 |
| Aspartate aminotransferase (U/liter) | 42.49 ± 0.76 | 41.82 ± 0.33 | 0.42 |
| Creatine kinase (U/liter) | 243.1 ± 3.28 | 239.5 ± 3.25 | 0.43 |
| PaCO2 (mmHg) | 41.68 ± 0.88 | 40.89 ± 0.70 | 0.48 |
| PaO2 (mmHg) | 94.75 ± 2.37 | 92.65 ± 1.99 | 0.50 |
| Age (year) | 52.45 ± 0.94 | 51.54 ± 0.99 | 0.50 |
| Systolic BP (mmHg) | 113.6 ± 1.12 | 112.5 ± 1.11 | 0.51 |
| Diastolic BP (mmHg) | 61.74 ± 0.80 | 61.08 ± 0.77 | 0.55 |
| Temperature (℃) | 37.22 ± 0.05 | 37.26 ± 0.05 | 0.56 |
| Male sex (no. /total no.) | 48.89% | 47.60% | 0.80 |
| Serum Glucose Lowest (mg/dL) | 124.4 ± 2.69 | 123.5 ± 2.56 | 0.80 |
| Serum Albumin Highest (g/dL) | 2.27 ± 0.04 | 2.26 ± 0.04 | 0.83 |
| Respiration (beats/min) | 24.8 ± 0.40 | 24.9 ± 0.44 | 0.86 |
| Serum Albumin Lowest (g/dL) | 2.33 ± 0.04 | 2.32 ± 0.04 | 0.88 |
| Urineout (ml) | 1662 ± 77.77 | 1676 ± 71.89 | 0.89 |
| Alanine aminotransferase (U/liter) | 49.02 ± 0.86 | 49.14 ± 0.60 | 0.91 |
| Heart rate (beats/min) | 95.36 ± 1.11 | 95.51 ± 1.20 | 0.93 |
| Shock (no. /total no.) | 50.92% | 51.66% | 0.93 |
| Predicted Body Weight (kg) | 62.56 ± 0.72 | 62.47 ± 0.65 | 0.93 |
| Height (cm) | 168.8 ± 0.69 | 168.8 ± 0.60 | 0.96 |
| Creatinine (mg/dl) | 1.537 ± 0.06 | 1.535 ± 0.08 | 0.98 |

e-Table 4. Baseline Characteristics of patients in phenotype1

| Characteristics | Placebo(n=111) | Rosuvastatin(n=136) | P |
| --- | --- | --- | --- |
| Age — yr | 54.35±16.39 | 53.84±17.03 | 0.81 |
| Male sex — no. /total no. | 46% | 49% | 0.70 |
| White race — no./total no. | 85% | 86% | 0.58 |
| Primary cause of lung injury  — no./total no. | 72% | 79% | 0.29 |
| Weight — kg | 86.68±29.11 | 85.40±27.62 | 0.72 |
| APACHE III score | 100.45±29.26 | 91.06±27.38 | 0.01 |
| Shock at baseline —no./total no. | 65% | 63% | 0.71 |
| Systolic BP (mmHg) | 109.10±19.89 | 110.32±18.26 | 0.62 |
| Diastolic BP (mmHg) | 60.74±11.93 | 60.54±11.67 | 0.89 |
| Tidal volume — ml/kg of predicted body weight | 6.66±0.12 | 6.59±0.09 | 0.61 |
| Minute ventilation — liters/min | 11.45±3.27 | 10.76±3.39 | 0.11 |
| Inspiratory plateau pressure  — cm of water | 14.86±4.59 | 14.23±4.30 | 0.26 |
| PEEP — cm of water | 8.82±3.58 | 8.76±3.38 | 0.89 |
| PaCO2 — mm Hg | 39.67±17.70 | 39.56±13.98 | 0.96 |
| PaO2:FiO2 — mm Hg | 141.98±66.61 | 137.98±57.88 | 0.61 |
| Creatinine — mg/dl | 1.53±0.86 | 1.74±1.53 | 0.20 |
| Creatine kinase — U/liter | 323.61±613.03 | 276.42±509.75 | 0.51 |
| Alanine aminotransferase  — U/liter | 43.60±46.69 | 41.40±31.94 | 0.66 |
| Aspartate aminotransferase  — U/liter | 63.27±59.13 | 60.93±43.16 | 0.72 |

e-Table 5. Baseline Characteristics of patients in phenotype2

| Characteristics | Placebo(n=126) | Rosuvastatin(n=118) | P |
| --- | --- | --- | --- |
| Age — yr | 52.73±15.48 | 55.00±18.41 | 0.30 |
| Male sex — no. /total no. | 51% | 51% | 1 |
| White race — no./total no. | 90% | 85% | 0.49 |
| Primary cause of lung injury  — no./total no. | 79% | 83% | 0.47 |
| Weight — kg | 91.52±35.04 | 93.08±34.59 | 0.73 |
| APACHE III score | 82.91±23.06 | 84.37±26.97 | 0.66 |
| Shock at baseline —no./total no. | 46% | 48% | 0.82 |
| Systolic BP (mmHg) | 116.02±17.36 | 113.32±19.61 | 0.25 |
| Diastolic BP (mmHg) | 62.11±14.09 | 60.58±13.77 | 0.39 |
| Tidal volume — ml/kg of predicted body weight | 8.23±1.78 | 6.64±0.11 | 0.39 |
| Minute ventilation — liters/min | 10.64±2.99 | 10.75±2.79 | 0.76 |
| Inspiratory plateau pressure  — cm of water | 15.32±4.34 | 15.40±5.19 | 0.91 |
| PEEP — cm of water | 9.48±4.38 | 9.48±4.11 | 0.99 |
| PaCO2 — mm Hg | 41.92±11.30 | 41.40±8.42 | 0.68 |
| PaO2:FiO2 — mm Hg | 144.82±58.04 | 152.72±66.58 | 0.32 |
| Creatinine — mg/dl | 1.53±1.20 | 1.41±0.97 | 0.38 |
| Creatine kinase — U/liter | 227.47±283.71 | 223.13±286.78 | 0.91 |
| Alanine aminotransferase  — U/liter | 33.84±27.80 | 29.32±23.92 | 0.18 |
| Aspartate aminotransferase  — U/liter | 44.95±33.97 | 38.86±22.86 | 0.10 |

e-Table 6. Baseline Characteristics of patients in phenotype3

| **Characteristics** | **Placebo(n=26)** | **Rosuvastatin(n=40)** | **P** |
| --- | --- | --- | --- |
| **Age — yr** | 53.88±15.59 | 54.93±17.47 | 0.81 |
| **Male — no. /total no.** | 58% | 53% | 0.87 |
| **White race — no./total no.** | 92% | 84% | 0.32 |
| **Primary cause of lung injury**  **— no./total no.** | 0.81 | 0.78 | 0.99 |
| **Weight — kg** | 98.46±34.59 | 89.20±27.61 | 0.23 |
| **APACHE III score** | 85.73±26.75 | 89±27.89 | 0.64 |
| **Shock at baseline —no./total no.** | 62% | 62% | 0.51 |
| **Systolic BP (mmHg)** | 119.96±20.98 | 11.80±17.78 | 0.09 |
| **Diastolic BP (mmHg)** | 64.58±18.01 | 58.50±10.67 | 0.09 |
| **Tidal volume — ml/kg of predicted body weight** | 6.79±0.32 | 6.48±0.18 | 0.37 |
| **Minute ventilation — liters/min** | 11.08±3.02 | 10.63±2.35 | 0.50 |
| **Inspiratory plateau pressure**  **— cm of water** | 15.19±4.45 | 15.55±4.13 | 0.74 |
| **PEEP — cm of water** | 9.46±3.86 | 9.70±3.39 | 0.79 |
| **PaCO2 — mm Hg** | 43.54±11.73 | 39.83±6.97 | 0.11 |
| **PaO2:FiO2 — mm Hg** | 137.08±65.15 | 140.70±66.47 | 0.83 |
| **Creatinine — mg/dl** | 1.58±1.45 | 1.33±0.76 | 0.36 |
| **Creatine kinase — U/liter** | 170±250.66 | 274.05±651.13 | 0.44 |
| **Alanine aminotransferase**  **— U/liter** | 28.92±22.38 | 32.65±26.04 | 0.55 |
| **Aspartate aminotransferase**  **— U/liter** | 40.50±24.51 | 45.30±34.34 | 0.54 |

e-Table 7. Baseline Characteristics of patients in phenotype4

| Characteristics | Placebo(n=18) | Rosuvastatin(n=10) | P |
| --- | --- | --- | --- |
| Age — yr | 57.67±14.48 | 55.20±13.51 | 0.66 |
| Male sex — no. /total no. | 39% | 70% | 0.24 |
| White race — no./total no. | 65% | 80% | 0.52 |
| Primary cause of lung injury  — no./total no. | 83% | 80% | 1 |
| Weight — kg | 85.17±26.45 | 91.3±33.23 | 0.60 |
| APACHE III score | 110.61±25.82 | 109.40±22.77 | 0.90 |
| Shock at baseline —no./total no. | 67% | 70% | 1 |
| Systolic BP (mmHg) | 108.50±19.17 | 107.40±20.079 | 0.89 |
| Diastolic BP (mmHg) | 54.78±10.38 | 56.40±10.63 | 0.70 |
| Tidal volume — ml/kg of predicted body weight | 6.95±0.36 | 7.43±0.84 | 0.55 |
| Minute ventilation — liters/min | 10.33±2.62 | 12±2.54 | 0.11 |
| Inspiratory plateau pressure  — cm of water | 13.78±5.60 | 14.80±3.55 | 0.61 |
| PEEP — cm of water | 9.11±3.36 | 8.20±2.66 | 0.47 |
| PaCO2 — mm Hg | 37.17±9.78 | 33.10±7.22 | 0.26 |
| PaO2:FiO2 — mm Hg | 138.61±88.89 | 110.60±47.54 | 0.37 |
| Creatinine — mg/dl | 1.94±0.94 | 2.8±1.75 | 0.10 |
| Creatine kinase — U/liter | 112.83±64.79 | 161.80±171.77 | 0.29 |
| Alanine aminotransferase  — U/liter | 42±33.83 | 32.7±20.89 | 0.44 |
| Aspartate aminotransferase  — U/liter | 55.56±43.51 | 60.70±34 | 0.75 |


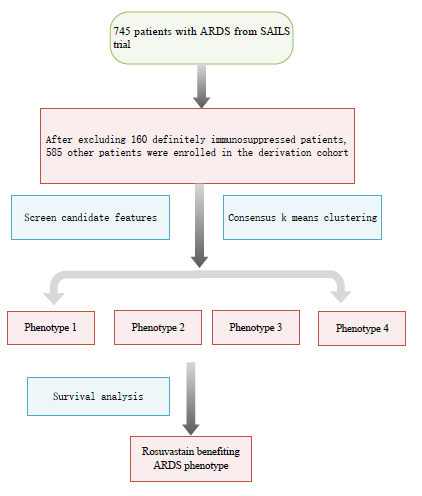


e-Figure1. The analysis flow plots of this study


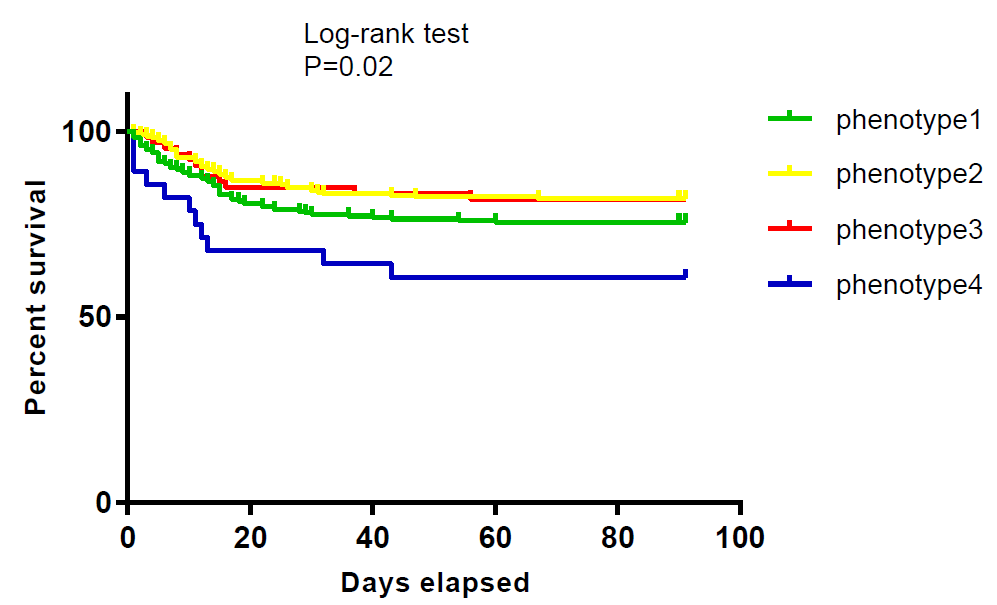


e-Figure 2. The survival curves of 4 phenotypes.


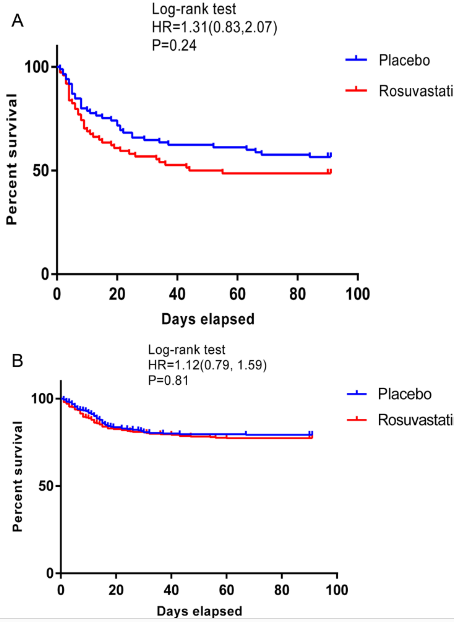


e-Figure 3. The survival curves of definitely immunosuppressed patients and other patients.

A. The Kaplan–Meier statistic analysis on definitely immunosuppressed patients.

B. The Kaplan–Meier statistic analysis on other patients.
